# Response of periodontal ligament stem cells to lipopolysaccharide and calcium silicate-based materials

**DOI:** 10.1590/0103-6440202204659

**Published:** 2022-04-29

**Authors:** Marlus da Silva Pedrosa, Handially dos Santos Vilela, Juliana Garuba Rahhal, Natália Pieretti Bueno, Fabianne Soares Lima, Fernando Neves Nogueira, Carla Renata Sipert

**Affiliations:** 1 University of São Paulo - USP, School of Dentistry, Department of Biomaterials and Oral Biology, São Paulo, SP, Brazil.; 2 University of São Paulo - USP, School of Dentistry, Department of Restorative Dentistry, São Paulo, SP, Brazil.; 3 University of São Paulo- USP, School of Dentistry, Department of Oral and Maxillofacial Surgery, Prosthesis and Traumatology, São Paulo, SP, Brazil.

**Keywords:** Endodontics, dental cements, periodontal ligament

## Abstract

This study was conducted to assess the *in vitro* response of human periodontal ligament stem cells (hPDLSCs) to bacterial lipopolysaccharide (LPS) activation and application of three calcium silicate-based materials (CSBM): Bio-C Sealer, MTA Fillapex and Cimmo HP. Characterization of the CSBM was performed by FTIR (n = 3). Extracts of Bio-C Sealer, MTA Fillapex and Cimmo HP were prepared and diluted (1:1, 1:4 and 1:16). Culture of hPDLSCs was established and treated or not with LPS from *Escherichia coli* (1 µg/mL) for 7 days. MTT assay was used to assess cell viability at 24, 48 and 72 h (n = 9). Alkaline phosphatase (ALP) activity was indirectly assayed at day 7 (n = 5). *TNF-α* and *Il* ^*-1*^ *0* cytokines were quantified by ELISA at 24h-cell supernatants (n = 6). Data were analyzed by ANOVA and Tukey’s test (α = 0.05). The cell viability of the LPS-activated hPDLSCs were higher than untreated control (p < 0.05). The application of CSBM affected the cell viability of untreated and LPS-activated cells (p < 0.05). ALP activity was higher for Bio-C Sealer and Cimmo HP in untreated and LPS-activated cells, respectively (p < 0.05). Application of CSBM normalized the *TNF-α* secretion in the LPS-activated cells (p < 0.05). Only MTA Fillapex in untreated hPDLSCs presented higher values of *Il* ^*-1*^ *0* (p < 0.05). Taken collectively, the results suggests that the simulation of the inflammatory process by LPS affect the *in vitro* response the hPDLSCs to the application of the CSBM.

## Resumo

Este estudo objetivou avaliar a resposta *in vitro* de células-tronco do ligamento periodontal humano (hPDLSCs) à ativação por lipopolissacarídeo bacteriano (LPS) e aplicação de três materiais à base de silicato de cálcio (CSBM): Bio-C Sealer, MTA Fillapex e Cimmo HP. A caracterização dos CSBM foi realizada por FTIR (n = 3). Extratos de Bio-C Sealer, MTA Fillapex e Cimmo HP foram preparados e diluídos (1:1, 1: 4 e 1:16). A cultura de hPDLSCs foi estabelecida e tratada ou não com 1 µg / mL de LPS de Escherichia coli por 7 dias. O ensaio de MTT foi usado para avaliar a viabilidade celular em 24, 48 e 72 h (n = 9). A atividade de ALP foi avaliada indiretamente no dia 7 (n = 5). As citocinas TNF-α e Il^-1^0 foram quantificadas por ELISA em sobrenadantes de células em 24h (n = 6). Os dados foram analisados por ANOVA e teste de Tukey (α = 0,05). A viabilidade celular das hPDLSCs ativados por LPS foi maior do que o controle (p <0,05). A aplicação dos CSBM afetou a viabilidade celular de células ativadas ou não por LPS (p <0,05). A atividade de ALP foi maior para Bio-C Sealer e Cimmo HP em células não ativadas e ativadas por LPS, respectivamente (p <0,05). A aplicação dos CSBM normalizou a secreção de TNF-α nas células ativadas por LPS (p <0,05). Apenas o MTA Fillapex em hPDLSCs não ativadas apresentou valores mais elevados de Il^-1^0 (p <0,05). Em conclusão, os resultados sugerem que a simulação do processo inflamatório por LPS afetou a resposta *in vitro* de células-tronco do ligamento periodontal e de materiais à base de silicato de cálcio.

## Introduction

Recent advances in dental materials have greatly improved the outcome and success rate of endodontic treatments. Compared to previous endodontic sealers and cements, calcium silicate-based materials (CSBM) are more biocompatible besides to stimulate hard tissue formation ^1^. In addition, CSBM present wide range of applications ^2^, being able to be used in pulp capping, pulpotomy, perforation repair, resorption defects, apexogenesis and as retrograde filling materials, apexification and as endodontic sealers ^2^.

During the endodontic treatment, despite the possibility of placing the CSBM in direct contact with stem cells, an influx of undifferentiated stem cells from periradicular tissues into the root canal may occur ^3^. Human periodontal ligament stem cells (hPDLSCs) reside in the perivascular space of the periodontium and are a promising tool for tissue regeneration ^2^. Interestingly, literature report that CSBM may provide a more favorable environment for periodontal ligament fibroblasts in root canal perforation ^4^ and consequently, tissue repair.

Lipopolysaccharide (LPS) is an endotoxin and the main constituent of gram-negative bacterial cell wall ^5^. Cytokines are small proteins released by cells, which are key modulators of inflammation. In contact with cells, LPS induces inflammatory and immune responses characterized by the release of a large number of cytokines including Tumor necrosis factor-α (TNF-α) ^6^ and Interleukin^-1^0 (IL^-1^0) ^7^ by Toll-like receptors ^8^ and the AKT kinase pathway ^7^, respectively. TNF-α is a pro-inflammatory cytokine that leads to various cellular responses including cell survival, differentiation, and proliferation ^9^. IL^-1^0 presents potent anti-inflammatory properties, being able to downregulate the production of inflammatory cytokines such as TNF-α.

Considering that root canal infection is predominantly rendered by gram-negative bacteria ^10^, LPS may be considered to simulate an inflammatory micro-environment in hPDLSCs *in vitro* ^11^. T Several literature reports focused on the *in vitro* cytotoxicity and osteogenic potential of CSBM on stem cells ^12^ ^;^ ^13^ ^;^ ^14^ ^;^ ^15^ ^;^ ^16^. However, the majority of these studies were mainly carried out without considering the inflammatory process caused by bacterial injury previous to the cell contact to materials ^12^ ^;^ ^13^ ^;^ ^14^ ^;^ ^15^ ^;^ ^16^. A previous study of our group demonstrated that priming cells with Enterococcus faecalis lipoteichoic acid significantly altered cellular viability to root canal dressings *in vitro* ^17^. In this study, we evaluated the potential response of hPDLSCs to three CSBM (Cimmo HP, Bio-C Sealer and MTA Fillapex) under a pre-stimulation with LPS. The null hypotheses tested were as follows: 1. the stimulation of inflammatory process by LPS would have no influence on cell viability, cytokine production and osteogenic potential of the hPDLSCs; 2. the cell viability, cytokine production and osteogenic potential would not be influenced by the application of the CSBM.

## Material and Methods

The experimental protocol was approved by the Ethics Committee of the School of Dentistry of the University of São Paulo (Protocol# 3.895.056, CAAE: 29154920.3.0000.0075 by Plataforma Brasil/CONEP) and was conducted in accordance with the Declaration of Helsinki.

### ATR-FTIR spectroscopy

The materials were manipulated following the manufacturer's instructions (Table 1) and inserted into a matrix designed for the production of specimens (7 mm x 1 mm, n = 3) and stored dry for 24 h (37 °C). After this period, the specimens had their surface evaluated by mid-infrared spectroscopy (Vertex 70, Bruker Optics GmbH, Germany) using an attenuated full reflectance accessory (ATR, MIRacle, Pike Technologies, Inc., Madison, WI, USA) with diamond crystal. The spectra were collected at three different points of the specimen in the range between 400 cm^-1^ to 4,000 cm^-1^ at a resolution of 4 cm^-1^, using 64 scans per spectrum.

### Culture of hPDLSCs

hPDLSCs previously characterized ^18^ were obtained from the cell biobank of the School of Dentistry of the University of São Paulo were cultured in proliferation medium (PM): α-MEM (Invitrogen - Thermo Fisher Scientific, Waltham, MA, USA) with 10 % fetal bovine serum (FBS) (Gibco - Thermo Fisher Scientific, Waltham, MA, USA) and antibiotics (100 µg/mL penicillin, 100 µg/mL streptomycin, 0.5 mg/mL amphotericin B - Invitrogen) at standard culture conditions (37 °C, 100 % humidity, 5 % CO2 and 95 % air). hPDLSCs cells from passages four to eight were used for the Assays. hPDLSCs cells were seeded at 1.25 x 10^4^ cells per well.

### Specimen and extract preparation

All materials (Table 1) were manipulated and inserted into a round metal appliance designed for the production of specimens (5 mm wide and 3 mm high). For Bio-C Sealer, a drop (60 uL) of distilled water was applied on the material to provide the moisture necessary for setting. Materials were allowed to set for 24 h in a humid atmosphere and aseptic conditions. After setting, each specimen was immersed into 1 mL of α-MEM with 10% FBS and incubated for 72 h. The specimens were then discarded and the extracts were filtered by 0.22-µm pore size membranes (Millipore; Billerica, MA, USA) ^19^ ^,^ ^20^ and stored at - 80 ºC until use. The extracts were diluted (1:1, 1:4, 1:16) in PM for MTT assays and osteogenic medium (OM) for alkaline phosphatase (ALP) activity assay. OM was prepared by adding 100 nM dexamethasone, 10 mM β-glycerol-phosphate, and 0.05 mM 2-phosphate-ascorbic acid into the PM.

### Treatment with lipopolysaccharide

The hPDLSCs were treated or not with 1 µg/mL of E. coli LPS (L4391; Sigma-Aldrich, St Louis, MO) for 7 days with medium change every other day. Next, cells were detached, counted and seeded.

### Cell viability assay

The untreated and LPS-activated hPDLSCs were counted and seeded at 1.25 ×10^4^ cells/well in 96-well plates in PM (n = 9). After 24 h, cells were incubated with 100 µL of the extracts dilutions for 24, 48 and 72 h. In the negative control group (NC), 100 µL of PM were applied to the cells ^20^. The medium was changed every two days. Then, the cell supernatant was replaced by 20 μL of a solution of 5 mg/mL of MTT (Sigma-Aldrich, St. Louis, MO, USA) in PBS 1X, followed by 180 μL of PM. Cells were incubated for 4 h and MTT solution was replaced by 100 µL of dimethyl sulfoxide (Synth, Diadema, SP, Brazil). Optical density was determined at 570 nm.

**Table 1:** Materials tested and manufacturer’s information

Materials	Manufacturer	Composition	Proportion
Bio-C Sealer	Angelus, Londrina, PR, Brazil	Calcium silicates, calcium aluminate, calcium oxide, zirconium oxide, iron oxide, silicon dioxide, dispersing agent	Ready to use / 60 uL of distilled water*
MTA Fillapex	Angelus, Londrina, PR, Brazil	Paste A: salicylate resin, bismuth trioxide, and fumed silica Paste B: fumed silica, titanium dioxide, mineral trioxide aggregate, and base resin	1g:1g (paste/paste)
Cimmo HP	Cimmo Soluções em Saúde, Pouso Alegre, MG, Brazil	Calcium oxide, calcium carbonate, magnesium oxide, dicalcium silicate, aluminum oxide, sodium oxide, potassium oxide and pozzolan with additives	0.2g powder/ 60uL liquid (distilled water)

### Alkaline phosphatase activity assay

The untreated and LPS-activated hPDLSCs were seeded in a 48-wells plate (2 × 10^4^ cells/well) and stimulated with 200 µL the extracts of the CSBM (n = 5) for 7 days. In the negative control group (NC), only OM were applied to the cells. The medium was changed every two days. The ALP activity was indirectly assayed using a kit (Labtest Diagnostica SA, Brazil). Briefly, the media were removed and 1% of sodium lauryl sulfate was added to each well. Then, 50 μL of the cell lysate, 50 μL of thymolphthalein monophosphate substrate and 500 μL of buffer were mixed and kept for 10 min 36 °C. The absorbance at 590 nm was measured (Synergy HT, Biotek, Instruments, Inc. Winooski, VT, USA). ALP activity was normalized by the total protein content and expressed as μmol of thymolphthalein/h/mg of protein.

### Quantification of cytokines

The untreated and LPS-activated hPDLSCs were culture (2 x 10^4^) in 96-well plates in PM and stimulated with the CSBM (1:4 dilution) for 24 h (n = 6). In the negative control group (NC), 100 µL of PM were applied to the cells. Quantification of TNF-α and IL^-1^0 concentrations were performed in the cell culture supernatants by commercially available Duo-Set Enzyme-linked immunosorbent assay (ELISA) kits from R & D Systems.

### Statistical analysis

Normal data distribution was verified through the Shapiro-Wilk normality test and data were analyzed by ANOVA and Tukey’s test (α = 0.05). Data are presented as mean ± standard deviation. All statistical analyses were performed using GraphPad Prism 7.00 (GraphPad Software, Inc., CA, US).

## Results

### ATR-FTIR spectroscopy

**Figure 1:**
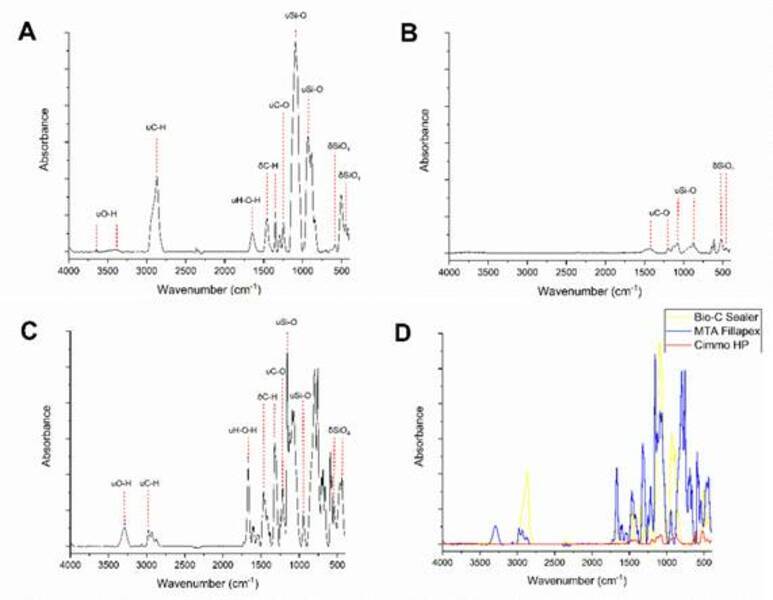
ATR-FTIR spectroscopy Bio-C Sealer (A), Cimmo HP (B), MTA Fillapex (C) and all (D). The main peaks are highlighted. The vibrational mode of the bonds is represented by δ (bending) and υ (stretching vibrations).

Small variations are observed between the CSBM, but in general there are: δSiO_4_ at 494 cm^-1^ and 573 cm^-1^, referring to dicalcium silicate and tricalcium silicate, respectively; υSi-O at 912 cm^-1^ and 1112 cm^-1^, coming from dicalcium silicate and tricalcium silicate, respectively; υC-O in 1246 cm^-1^, δC-H in 1348 cm^-1^ and 1456 cm^-1^ and υC-H in 2868 cm^-1^, from polyethylene glycol; υH-O-H in 1647 cm^-1^, corresponding to the hydrated phase of cement (calcium silicate hydrate); υO-H in 3431 cm^-1^ and 3637 cm^-1^, referring to the hydrated and non-hydrated phase (calcium hydroxide) of cement, respectively ^21^ ^,^ ^22^ (Figure 1).

### Cell viability assay

**Figure 2:**
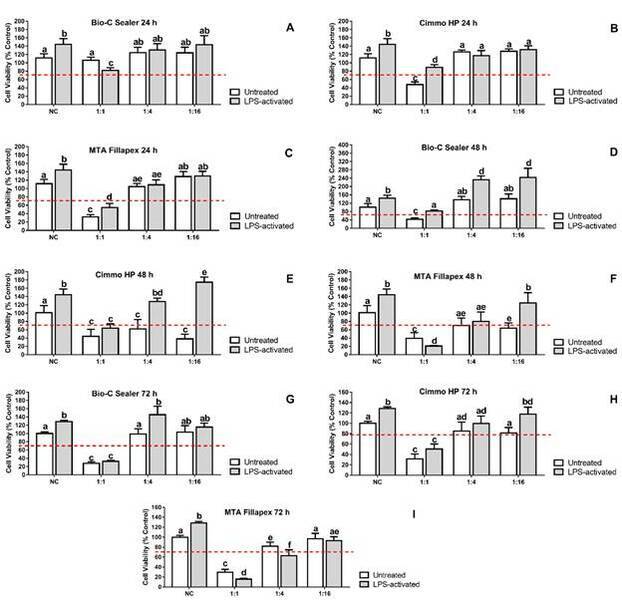
Cell viability rate (% of negative control group) according to MTT assay in hPDLSCs after 24 (A, B and C), 48 (D, E and F) and 72 (G, H and I) hours of exposure to different dilutions (1:1, 1:4 and 1:16) of the sealer extracts of Bio-C Sealer (A, D and G), Cimmo HP (B, E and H) and MTA Fillapex (C, F and I). hPDLSCs incubated in culture medium alone served as the negative control group. hPDLSCs were seeded in at a cell density of 2x10^4^ cells well in a 96-well culture plate. The results show mean and standard deviation of the experiments (n = 9). Different letters represent signiﬁcant differences between groups. Two-Way ANOVA with Tukey test (p < 0.05). The horizontal dashed line indicate 70% cell viability.

The cell viability of the LPS-activated hPDLSCs were higher than untreated control in all experimental periods (Figure 2).

The pure extract (1:1) of Bio-C Sealer at 24 h (Figure 2A) for LPS-activated hPDLSCs and 48 (Figure 2B) and 72 h (Figure 2C) for LPS-activated and untreated cells were cytotoxic (p < 0.05). At 48 h, higher cell viability was observed for 1:4 and 1:16 dilutions (p < 0.05). Interestingly, the cell viability in untreated cells were lower than the LPS-activated ones (p < 0.05). At 72 h, higher cell viability for 1:4 dilution of Bio-C Sealer in the LPS-activated compared to untreated cells was observed (p < 0.05).

A reduction in cell viability was observed for undiluted Cimmo HP at 24 (Figure 2D), 48 (Figure 2E) and 72 h (Figure 2F) (p < 0.05). This reduction was only cytotoxic in untreated cells at 24 h. At 48 h, only 1:4 and 1:16 dilutions of Cimmo HP in LPS-activated cells were not cytotoxic (p < 0.05).

The pure extract (1:1) of MTA Fillapex was cytotoxic in untreated LPS-activated cells at 24 (Figure 2G), 48 (Figure 2H) and 72 h (Figure 2I) (p < 0.05).

### Alkaline phosphatase activity

**Figure 3:**
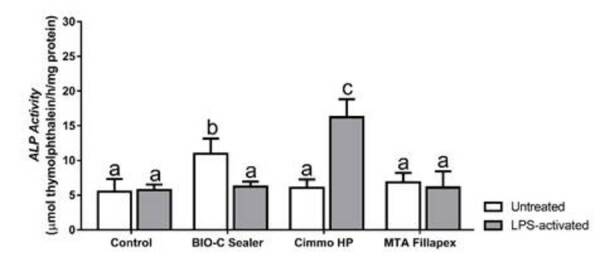
ALP activity after 7 days of exposure to 1:16 dilution of the extracts of Bio-C Sealer, Cimmo HP and MTA Fillapex in untreated and LPS-treated hPDLSCs. hPDLSCs incubated in culture medium alone served as the negative control. The results show mean and standard deviation of the experiments (n = 5). Different letters represent signiﬁcant differences between groups. Two-Way ANOVA with Tukey test (p < 0.05).

The ALP activity (Figure 3) was significantly higher for Bio-C Sealer and Cimmo HP in untreated and LPS-activated hPDLSCs, respectively (p < 0.05).

### Cytokine production

**Figure 4:**
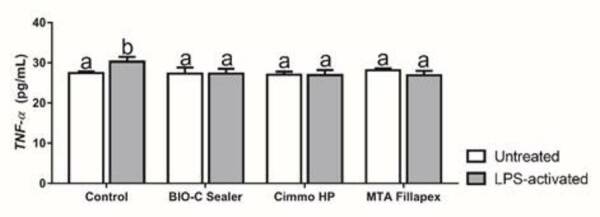
*TNF-α* concentration according to ELISA assay after 24 h of exposure to 1:4 dilution of the extracts of Bio-C Sealer, Cimmo HP and MTA Fillapex in untreated and LPS-treated hPDLSCs. hPDLSCs incubated in culture medium alone served as the negative control. The results show mean and standard deviation of the experiments (n = 6). Different letters represent signiﬁcant differences between groups. Two-Way ANOVA with Tukey test (p < 0.05).

**Figure 5:**
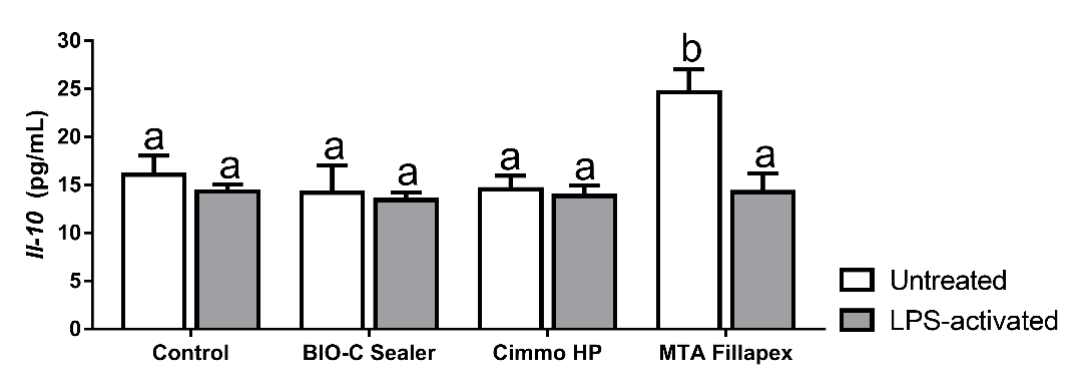
*Il* ^*-1*^ *0* concentration according to ELISA assay after 24 h of exposure to 1:4 dilution of the extracts of Bio-C Sealer, PBS Cimmo HP and MTA Fillapex in untreated and LPS-treated hPDLSCs. hPDLSCs incubated in culture medium alone served as the negative control. The results show mean and standard deviation of the experiments (n = 6). Different letters represent signiﬁcant differences between groups. Two-Way ANOVA with Tukey test (p < 0.05).

The concentration of TNF-α (Figure 4) was significantly higher for LPS-activated control group (p < 0.05). No differences were observed in TNF-α concentration between the CSBM and untreated negative control (p > 0.05). Contrastingly, for IL^-1^0 (Figure 5), only MTA Fillapex in untreated hPDLSCs presented higher values compared to control, Bio-C Sealer and Cimmo HP (p < 0.05). These values were lower in the LPS-activated cells (p > 0.05).

## Discussion

Calcium silicate-based materials are able to affect the cellular behavior of stem cells and consequently affect wound healing events and tissue repair ^2^. In this study, the null hypotheses were rejected. The results showed that the pre-activation with LPS significantly affected the cellular response to CSBM considering cell viability and/or proliferation, alkaline phosphatase activity and cytokines production by hPDLSCs .

The intensity of the peaks presented in the FTIR-ATR analysis indicate that the amount of molecules available in the calcium silicate-based materials after setting were mainly dicalcium and tricalcium silicate, calcium silicate hydrate, and calcium hydroxide ^21^ ^,^ ^22^. The highest vibrational modes indicate higher amount of molecules available ^21^ ^,^ ^22^. In this study, highest vibrational modes were found for Bio-C Sealer and MTA Fillapex. The peak characteristic of the hydrated phase of CSBM was not observed for Cimmo HP, which suggests that the setting reaction was not fully completed. Consequently, this could affect the solubility and leaching of the components of CSBM into the extract and consequently have influence on the biological results investigated here. These data should be complemented by further analysis of the ions released from the CSBM into the culture medium.

In this study, different dilutions of the CSBM extracts were assessed to investigate the role of releasing components that potentially diffuse into the living tissues in vivo ^19^ ^,^ ^23^. MTT assay was used to assess cell viability according to ISO 10993-5:1999 recommendations. This method is considered reliable for the cytotoxicity screening of endodontic materials as it does not cause over- or underestimation of cell viability ^24^. CSBM may lead to different responses depending on the cell line ^25^. hPDLSCs were chosen for this study since they are present on the surface of the tooth and boundary locations of the periodontium and are important for apical tissue regeneration and inflammatory response ^26^.

According to ISO 10993-5:1999, biomaterials that cause a reduction in cell viability by more than 30% are considered cytotoxic ^27^. In this study, BIO-C sealer, CIMMO and MTA-Fillapex presented mild and transient cytotoxicity at untreated cells corroborating a previous study ^18^. Interestingly, LPS activation resulted in increased proliferation for cells treated with Bio-C sealer and CIMMO, but not with MTA-Fillapex. As previously observed for LTA ^17^, we may speculate that since absorbance exceeded 100%, LPS induced cell proliferation that in turn is differently affected by each material tested. For the present study, TNF-α and IL^-1^0 may not justify these differences observed since their production was not correlated to these events.

When placed in contact with LPS-activated hPDLSCs, higher cell viability was found for some dilutions of the CSBM. Induced inflammation with LPS from Porphyromonas gingivalis (P. gingivalis LPS, 10 ng / mL for 24 h) was shown to reduce proliferation and migration of hPDLSCs ^11^. P. gingivalis LPS differs from E. coli LPS in its structure and functional activity ^28^. This might lead to distinct biological events such as quantification of cytokines and of alkaline phosphatase activity. In the present study, the choice for E. coli LPS is based on the structural and biological similarity to LPS from Fusobacterium nucleatum, which is one of the most prevalent bacteria found in endodontic infections ^29^ ^,^ ^30^. As observed herein with E. coli LPS, literature reports that LPS-activation exert protective effects against cytotoxic effects in PC12 Cell Lines. In addition, LTA-primed apical papilla cells presented increased cell proliferation solely ^17^ but increased loss of viability for intracanal dressings.

It should be emphasized that alkaline phosphatase is a marker of mineralization associated with odontogenic differentiation ^31^. To determine whether the CSBM would favor osteogenic differentiation *in vitro*, ALP activity was measured at day 7. Only Bio-C Sealer in untreated hPDLSCs showed increased ALP activity compared to control, which in turn is downregulated by LPS-activation. On the other hand, for Cimmo HP, increased ALP activity was found only when this material was placed in contact with LPS-activated hPDLSCs. As these materials are relatively new, literature lacks on studies regarding their effects on ALP activity that could led to a better discussion of our results. However, as observed here, the application of CSBM on hPDLSCs led to increased ALP activity ^32^.

TNF-α is an important pro-inflammatory cytokine ^9^. In our study, LPS-activation increased TNF-α secretion by hPDLSCs that in turn was abrogated by the three tested materials. In fact, LPS-induced activation of hPDLSCs causes the release of cytokines, including TNF-α ^33^. In untreated hPDLSCs, the application of CSBM neither induced nor aggravated TNF-α secretion. In a previous study ^18^, we verified that the application of Bio-C Sealer, Cimmo HP and MTA Fillapex in untreated hPDLSCs lead to the release of TNF-α, suggesting an inflammatory potential when in contact with living cells. The observed differences might be due to the different dilution used for the cytokine quantification as well as the experimental period.

The observation that the secretion of TNF-α by LPS-activated hPDLSCs was normalized by the application of the CSBM lead to the assumption that these materials could have a positive effect in modulating the inflammatory process caused by the prior exposure to LPS. In untreated C3T3-E1 ^34^ and hPDLSCs ^16^ stimulated with CSBM, a reduction of the expression of TNF-α was found ^16^. To the best of our knowledge, relevant information regarding TNF-α secretion by LPS-activated hPDLSCs stimulated with Bio-C Sealer, Cimmo HP, and MTA Fillapex are missing in the current literature.

IL^-1^0 is an anti-inflammatory cytokine that exerts an important role in the specific and unspecific immune reactions and consequently, in tissue damage ^9^. Besides the literature reports a decreased in IL^-1^0 secretion by hPDLSCs under challenge P. gingivalis LPS (5 ug/ml) ^33^, our results showed no difference. This probably due to the differences in the types and concentrations of LPS used as well as the periods of treatments. In addition, only MTA Fillapex increased IL^-1^0 secretion in untreated hPDLSCs, which was decreased with in the LPS-activated cells. This increased in untreated cells was also observed in a previous study ^18^.

As *in vitro* studies present limitations, which were previously described in the scientific literature ^35^, the results reported herein should be interpreted with caution. In addition, the extrapolation of *in vitro* results to clinical practice has been a great challenge. However, besides the results of this study should be complemented by further *in vitro* and in vivo studies including longer periods of evaluation, they allow a preliminary understanding of the behavior of these endodontic materials in a more clinical scenario, which is of great interest for clinical practice.

In conclusion, the results suggest that the simulation of the inflammatory process by LPS affects *in vitro* response of the hPDLSCs to the application of the CSBM. In general, Bio-C sealer, CIMMO and MTA-Fillapex showed proliferation induction at LPS-treated cells. ALP activity induced by Bio-C sealer was abrogated in LPS-activated cells while increased by CIMMO only at LPS-treated cells. These events were not dependent on TNF-α or IL^-1^0 production. This also calls attention to the need of more *in vitro* studies considering the inflammatory process caused by traumatic or bacterial injury to assess the performance of endodontic materials *in vitro*.
